# The effect of cocoa supplementation on hepatic steatosis, reactive oxygen species and LFABP in a rat model of NASH

**DOI:** 10.1186/1476-5926-10-10

**Published:** 2011-11-14

**Authors:** Mile Janevski, Kiriakos N Antonas, Melanie J Sullivan-Gunn, Maree A McGlynn, Paul A Lewandowski

**Affiliations:** 1 School of Medicine, Deakin University, Waurn Ponds, Australia; 2 School of Biomedical Science, Victoria University, Melbourne, Australia; 3 School of Sport and Science, Victoria University, Melbourne, Australia

**Keywords:** Non alcoholic steatohepatitis, NASH, oxidative stress, antioxidant, liver fatty acid binding protein, NADPH oxidase, cocoa

## Abstract

**Background:**

Non alcoholic steatohepatitis is hypothesised to develop via a mechanism involving fat accumulation and oxidative stress. The current study aimed to investigate if an increase in oxidative stress was associated with changes in the expression of liver fatty acid binding protein in a rat model of non alcoholic steatohepatitis and whether cocoa supplementation attenuated those changes.

**Methods:**

Female Sprague Dawley rats were fed a high fat control diet, a high fat methionine choline deficient diet, or one of four 12.5% cocoa supplementation regimes in combination with the high fat methionine choline deficient diet.

**Results:**

Liver fatty acid binding protein mRNA and protein levels were reduced in the liver of animals with fatty liver disease when compared to controls. Increased hepatic fat content was accompanied by higher levels of oxidative stress in animals with fatty liver disease when compared to controls. An inverse association was found between the levels of hepatic liver fatty acid binding protein and the level of hepatic oxidative stress in fatty liver disease. Elevated NADPH oxidase protein levels were detected in the liver of animals with increased severity in inflammation and fibrosis. Cocoa supplementation was associated with partial attenuation of these pathological changes, although the severity of liver disease induced by the methionine choline deficient diet prevented complete reversal of any disease associated changes. Red blood cell glutathione was increased by cocoa supplementation, whereas liver glutathione was reduced by cocoa compared to methionine choline deficient diet fed animals.

**Conclusion:**

These findings suggest a potential role for liver fatty acid binding protein and NADPH oxidase in the development of non alcoholic steatohepatitis. Furthermore, cocoa supplementation may have be of therapeutic benefit in less sever forms of NASH.

## Background

Non alcoholic fatty liver disease (NAFLD) involves a spectrum of conditions ranging from simple fat accumulation in the liver to end stage liver failure and cirrhosis. NAFLD can lead into the development of non alcoholic steatohepatitis (NASH) [1]. NASH is an emerging health concern and it is believed that its prevalence is on the rise due to escalating obesity rates [2]. Estimated NAFLD prevalence in Western countries is between 17-33% [3]. NAFLD accounts for up to 20%, and NASH accounts for 2-3% of liver test abnormalities in most developed countries [4].

NASH is typically reported in obese individuals suffering from one or a combination of type 2 diabetes, insulin resistance and dyslipidaemia, but is not restricted to this group [2]. There is often an increase in aspartate aminotransferase (AST) and alanine aminotransferase (ALT) [5]. Lipid accumulation occurs early in NASH as part of the development of the disease [6]. The two hit disease model postulates that steatosis is a trigger for the establishment of NASH and the increased levels of fat infiltration cause damage to the liver by forming fat droplets within the hepatic tissue, thus setting off the second hit of the disease by causing lipotoxicity. In addition, cytokines and reactive oxygen species (ROS) create a pro-oxidant state that can activate stellate cells to produce fibrotic scar tissue [7].

Liver fatty acid binding protein (LFABP) accounts for 3-5% of the cytosolic protein content in hepatocytes. LFABP is transcriptionaly regulated by the nuclear hormone receptor, peroxisome proliferator-activated protein α (PPAR-α), and is responsible for intracellular trafficking of long chain fatty acids [8]. Rat LFABP has recently been described as an endogenous antioxidant [9], and may be useful in states of extreme oxidative stress when intracellular antioxidants such as superoxide dismutase, glutathione and catalase cannot quench excessive quantities of ROS. This antioxidant characteristic of LFABP is thought to result from the methionine groups located in the cavity of the LFABP binding site [9].

NADPH oxidase (NOX), an enzyme complex responsible for generating superoxide, is activated in rat Kupffer cells in alcoholic liver disease, through induction of transcription factor NF-κβ and TNF-α production [10]. However, administration of a methionine choline deficient (MCD) diet to p47 knockout mice, lacking a critical subunit of the NOX complex, showed that NOX is not an important contributor of oxidative stress generation. The p47 knockout mice on an MCD diet developed NASH with similar pathology as wild type, despite the lack of a functional NOX enzyme [11].

Currently the only effective therapy to treat NAFLD and NASH is to decrease body weight in obese and overweight individuals, decrease circulating lipids, improve insulin sensitivity and decrease oxidative damage through the use of antioxidants [12]. Cocoa and some of its derivatives are a rich source of the flavonoid antioxidants, catechin and epicatechin [13]. In a high fat diet model of obesity, rats supplemented with cocoa had normalised insulin resistance and decreased weight gain. Furthermore, cocoa supplementation decreased gene expression of fatty acid binding protein in mesenteric adipose tissue [14]. Consumption of dark chocolate by human subjects for 15 days has been reported to improve blood pressure and insulin sensitivity [15]. Cocoa supplementation has been found to have a beneficial effect in a rat model of alcoholic steatohepatitis by reducing hepatic fat accumulation, inflammation and necrosis [16].

The current study aimed to investigate if an increase in oxidative stress was associated with changes in the expression of LFABP and NOX in a rat model of non alcoholic steatohepatitis and whether cocoa supplementation attenuated those changes.

## Methods

### Animals and diet

All animal experiments and procedures were approved by the animal welfare committee at Deakin University, approval number A36/2007. Twelve week old female Sprague Dawley rats (n = 56, Animal Resources Centre, Perth, Australia) were housed in pairs with ad libitum access to food and water. Female rats were selected to minimise fighting within pairs throughout the study. Three isocalorically matched diets were used in these investigations (Table 1). A high fat methionine choline sufficient (MCS) diet (control); a high fat methionine choline deficient (MCD) diet; and a high fat methionine choline deficient diet supplemented with 12.5% cocoa powder (MCS: A02082003B; MCD: A02082002B; Research Diets, New Brunswick, USA). The cocoa powder (Natraceutical, Valencia, Spain) contained 12% polyphenols, primarily catechin, and trace amounts of methionine (0.28 mg/g diet) and choline (0.02 mg/g diet). The MCD diet is a commonly used model of NASH and is known to cause weight loss [7]. A pilot study demonstrated that a period of 52 days was a suitable time frame to induce NAFLD, based on histological grading, and still maintain the body weight of rats fed the MCD diet. The pilot study indicated that histologically the livers of rats fed the MCD diet were the same after 42 days of feeding through to 112 days of feeding. Rats were divided into six groups (Table 2) and were fed either a MCS or MCD diet for 52 days or one of four cocoa supplementation regimes: 52 days of MCD and an additional 28 days of MCD with cocoa supplementation (C1); 52 days of MCD and an additional 56 days of MCD with cocoa supplementation (C2); 80 days of MCD with cocoa supplementation (C3); 108 days of MCD with cocoa supplementation (C4). The four feeding regimes were selected to represent treatment or prevention supplementation modes that could be applied to NASH patients. Feeding regimes C1 and C2 were used to test if cocoa supplementation for four or eight weeks could be used to treat NASH after the disease was established. Whereas, feeding regimes C3 and C4 were used to see if cocoa supplementation could be used to prevent or slow the development of NASH over the same total time periods used in regimes C1 and C2.

**Table 1:** Diet composition

Catalogue number	A02082002B	A02082003B	A07071301
Ingredients (g)	MCD	MCS	Cocoa (C1 - C4)
Protein	17	17.2	17
Carbohydrate	65.9	65.5	65.9
Fat	9.9	9.9	9.9
L-Alanine	3.5	3.5	2.9
L-Arginine	12.1	12.1	9.9
L-Asparagine-H_2_O	6	6	4.9
L-Aspartate	3.5	3.5	2.9
L-Cystine	3.5	3.5	2.9
L-Glutamine	40	40	32.8
Glycine	23.3	23.3	19.1
L-Histidine-HCl-H_2_O	4.5	4.5	3.7
L-Isoleucine	8.2	8.2	6.7
L-Leucine	11.1	11.1	9.1
L-Lysine-HCl	18	18	14.7
L-Phenylalanine	7.5	7.5	6.1
L-Proline	3.5	3.5	2.9
L-Serine	3.5	3.5	2.9
L-Threonine	8.2	8.2	6.7
L-Tryptophan	1.8	1.8	1.5
L-Tyrosine	5	5	4.1
L-Valine	8.2	8.2	6.7
Total L-Amino Acids	171.4	171.4	140.5
Sucrose	455.3	452.3	455.3
Corn starch	150	150	106
Maltodextrin	50	50	50
Cellulose	30	30	0
Corn oil	100	100	86
Mineral mix S10001	35	35	35
Sodium bicarbonate	7.5	7.5	7.5
Vitamin mix V10001	10	10	10
DL-Methionine	0	3	0.2*
Choline bitrate	0	2	0.017*
Cocoa powder	0	0	144
Total	1009.2	1011.2	1034.3

High fat methionine choline sufficient (MCS) diet, high fat methionine choline deficient (MCD) diet, high fat methionine choline deficient diet with 28 days of cocoa supplementation (C1), high fat methionine choline deficient diet with 56 days of cocoa supplementation (C2), high fat methionine choline deficient diet supplemented with cocoa for 80 days (C3) and high fat methionine choline deficient diet supplemented with cocoa for 108 days (C4).

* Derived from cocoa powder.

**Table 2:** Experimental groups, diets and duration of each diet regime

Diet	Diet regimes	MCS duration (days)	MCD duration (days)	MCD and cocoa duration (days)
MCS	High fat MCS	52	-	-
MCD	High fat MCD	-	52	-
C1	High fat MCD followed by 28 day cocoa supplementation	-	52	28
C2	High fat MCD followed by 56 day cocoa supplementation	-	52	56
C3	High fat MCD with cocoa supplementation	-	-	80
C4	High fat MCD with cocoa supplementation	-	-	108

High fat methionine choline sufficient (MCS) diet, high fat methionine choline deficient (MCD) diet, high fat methionine choline deficient diet with 28 days of cocoa supplementation (C1), high fat methionine choline deficient diet with 56 days of cocoa supplementation (C2), high fat methionine choline deficient diet supplemented with cocoa for 80 days (C3) and high fat methionine choline deficient diet supplemented with cocoa for 108 days (C4).

At the conclusion of each regime, animals were fasted overnight and euthanized at 8 am via a lethal dose of anaesthetic (70 mg/kg Lethabarb, Therapon, Melbourne, Australia). Blood samples were collected via cardiac puncture and the liver, heart, kidneys and pancreas removed and weighed. One lobe of the liver was fixed in 4% paraformaldehyde (Sigma, Sydney, Australia) and the remaining portion snap frozen and stored at -80°C for further analysis.

### Histological analysis

The liver histology was assessed on de-identified slides by two independent blinded observers after an initial consensus meeting. Haematoxylin and eosin (H&E) (Thermo Scientific, Melbourne, Australia) stained sections were scored for steatosis (0-3) and lobular inflammation (0-3) according to the revised Kleiner method [17]. The presence or absence of portal inflammation was also noted (0-1). Fibrosis was graded (0-4) using Sirius Red (Sigma, Sydney, Australia) stained sections [17]. A random subset of 10% of cases was rescored by each observer. Each animal had duplicate histological specimens prepared, and where scores differed between duplicates, the slides were rescored for consensus.

### Biochemical parameters and measures of oxidative stress

Plasma triglycerides and glucose levels were determined using appropriate assay kits according to the manufacturer's instructions (Thermo Scientific, Melbourne, Australia). Red blood cell (RBC) and liver tissue glutathione (GSH), an endogenous antioxidant, was measured via the GSH recycling method as previously described [18]. Briefly, RBC were obtained by centrifugation of blood (1000 × *g*) and 200 μl of RBC was used; 1 g of liver was homogenized. A change in absorbance (412nm) was determined after the addition of 5,5'-Dithiobis(2-nitrobenzoic acid) (Sigma, Sydney, Australia) and corrected to reduced L-glutathione standard (Sigma, Sydney, Australia). Liver GSH was corrected for protein concentration which was determined via the Bradford method (BioRad, Sydney, Australia). Dihydroethidium (DHE) staining (Sigma, Sydney, Australia) was used to detect levels of superoxide in liver cryosections (14 μm) [19]. DHE fluorescence was quantified using a fluorescence quantification program, ImageJ (National Institutes of Health, USA), as a measure of superoxide levels present in tissue. An ELISA kit was used to measure the DNA oxidation byproduct 8-hydroxy-2-deoxy guanosine (8-OH-2dG) (StressMarq Biosciences). DNA was extracted from 15 mg of liver tissue using a DNA isolation kit (Promega, Sydney, Australia). Each sample was then diluted so that 50 μg of DNA was used in the 8-OH-2dG assay. The competitive immunoassay involves the binding of free 8-OH-2dG to an antibody coated 96 well plate. The assay and sample concentration of 8-OH-2dG were carried out as per the manufacturer's instructions. Total 8-isoprostane concentrations were analysed as a marker of oxidative damage to lipids in liver homogenates prepared for liver GSH using an enzyme immunoassay (EIA) kit (Cayman Chemicals, Sydney, Australia) following manufactures instructions. Prior to analysis liver homogenates were hydrolysed by addition of 25 μl 2 M NaOH to each 100 μl homogenate. The samples were incubated at 45°C for 2 hours. Following this, 25 μl 10N HCl acid was added and the samples were centrifuged for 5 minutes at 12,000 *g*. The supernatant was removed and used for the determination of total 8-isoprostane using the EIA kit. The assay was based on the competition between 8-isoprostane and an 8-isoprostane acetycholinesterase (AChE) conjugate for a limited number of 8-iso-PGF2α-specific rabbit anti-serum binding sites, values were expressed as pg/mg of protein.

### RT-PCR

Total RNA was extracted from 50 mg of frozen liver using TRI reagent (Astral Scientific, Sydney, Australia) according to the manufacturer's specification. The total RNA concentration was determined by A260/A280 measurement. One microgram of total RNA was reverse transcribed into cDNA using AMV reverse transcriptase first strand cDNA synthesis kit according to the manufacturer's protocol (Marligen Biosciences, Sydney, Australia). Primers were designed using Primer3. Forward and reverse primer sequences are shown in Table 3. β-actin mRNA was quantified and showed no significant variation between feeding regimes, and all results were normalised to these values. The amplification of cDNA samples was carried out using IQ SYBR green™ following the manufacturers protocols (BioRad, Sydney, Australia) Fluorescent emission data was captured and mRNA levels were analyzed using the critical threshold (CT) value [20].Thermal cycling and fluorescence detection were conducted using the Biorad IQ50 sequence detection system (BioRad, Sydney, Australia).

**Table 3:** Primer sequences

Target	Sequence
β-actin	Forward- TGT CAC CAA CTG GGA CGA TA Reverse- AAC ACA GCC TGG ATG GCT AC
LFABP	Forward- CAT CCA GAA AGG GAA GGA CA Reverse- CAC GGA CTT TAT GCC TTT GAA
NOX1	Forward- TAC GAA GTG GCT GTA CTG GTT G Reverse- CTC CCA AAG GAG GTT TTC TGT T
NOX2	Forward- TCA AGT GTC CCC AGG TAT CC Reverse- CTT CAC TGG CTG TAC CAA AGG
NOX4	Forward- GGA AGT CCA TTT GAG GAG TCA C Reverse- TGG ATG TTC ACA AAG TCA GGT C

### Protein extraction and western blot analysis

Liver samples (100 mg) were homogenized and centrifuged at 10,000 *g *at 4°C for 10 minutes. The protein concentration was determined via the Bradford method (BioRad, Sydney, Australia); protein samples (10 μg) were separated via SDS-PAGE on a 4-20% gradient gel (NuSep, Sydney, Australia) and transferred onto polyvinylidene difluoride membranes. The membranes were treated as previously described [21]. Proteins were visualised using Immune-Star HRP substrate kit (BioRad, Sydney, Australia). The density of the bands was quantified using a Chemidoc system (BioRad, Sydney, Australia) and normalised to β-actin expression. LFABP primary antibody used was a rabbit polyclonal antibody (1:200). NOX1 primary antibody used was a rabbit polyclonal antibody (1:200). Secondary antibody used for both LFABP and NOX1 was a goat anti-rabbit IgG-HRP conjugated antibody (1:5000). β-actin primary antibody, mouse anti β-actin (1:200) and secondary goat anti mouse antibody (1:2000) were used. Antibodies were purchased from Santa Cruz Biotechnology (CA, USA).

### Statistical analysis

Statistical analysis was performed using SPSS analysis package (version 17.0), with independent t-tests and ANOVA with Tukey post-hoc analysis. The categorical assignments of the various histological aspects of NASH were statistically analysed by Fishers Exact test. Values were expressed as mean ± SEM and considered statistically significant with a *p*≤0.05.

## Results

### Histological analysis

**Figure 1:**
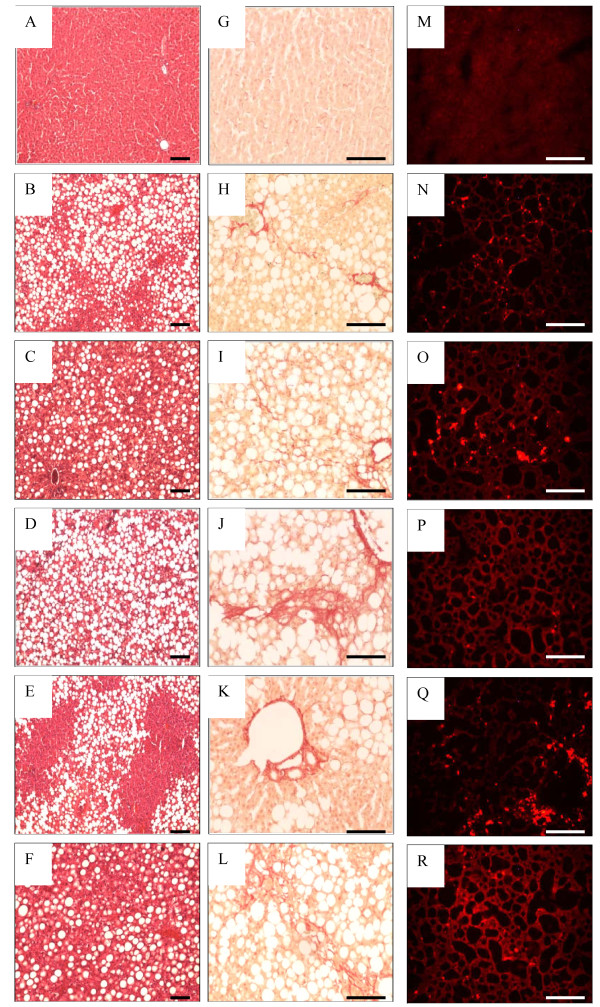
**Histological examination of liver sections by H&E stain (at left), Sirius Red stain (at middle) and DHE stain (at right)**. H&E stain (A-F): MCS diet (A), MCD diet (B), C1 (C), C2 (D), C3 (E), C4 (F). Sirius Red stain of fibrosis (G-L): MCS diet (G), MCD diet (H), C1 (I), C2 (J), C3 (K), C4 (L). DHE stain of superoxide (M-R): MCS diet (M), MCD diet (N), C1 (O), C2 (P), C3 (Q), C4 (R). Bar = 100 μm.

The results of the scoring of each of the histological variables for each of the groups are presented as percentages in Table 4. Histological analysis showed very little difference in observed steatosis or fibrosis between most of the groups (Table 4), with a few notable exceptions. A statistically significant higher steatosis score was seen in livers from animals fed the MCD diet compared to animals fed the MCS diet - which showed no or minimal steatosis, scoring 0 (Table 4*p *< 0.001). This high steatosis score seen with the pure MCD diet was also present in each of the cocoa supplemented diet regimes (again statistically different when compared to the MCS group, Table 4*p *< 0.001), with the exception of the C3 diet regime - the livers of which showed a lesser degree of steatosis when compared to the C1 and C2 diet regimes (Table 4*p *= 0.007). The presence of portal inflammation largely paralleled the degree of portal fibrosis, and each of these was most pronounced in the C2 group (Table 4 p < 0.001). Lobular inflammation was seen across the board in the MCD and cocoa supplemented diets to a relatively similar degree, but there was only weak statistical significance in this observation for some of the groups when compared to the degree of lobular inflammation in the MCS group (Table 4 p < 0.05). The lowest fibrosis scores were seen in the MCS group (Table 4 p < 0.05), and compared to the other cocoa supplementation groups, the livers from the animals on the C3 diet had the lowest fibrosis scores (Table 4 p < 0.05). Cirrhosis (Fibrosis score 4) was not seen in any of the livers from any of the animals in this study (Figure 1; Table 4).

**Table 4:** NASH scoring of H&E stained liver sections and fibrosis scores in Sirius Red stained liver sections

	Score	MCS (% of cases)	MCD (% of cases)	C1 (% of cases)	C2 (% of cases)	C3 (% of cases)	C4 (% of cases)
Steatosis	0	100%	0%	0%	0%	0%	0%
	1	0%	13%	0%	0%	31%	12%
	2	0%	17%	0%	0%	50%	19%
	3	0%	70%	100%	100%	19%	69%
Significant		MCD, C1, C2, C3, C4	MCS, C3	MCS, C3	MCS, C3	MCS, MCD, C1, C2	MCS
Portal inflammation	0	88%	83%	69%	21%	94%	94%
	1	12%	17%	31%	79%	6%	6%
Significant		C2	C2	C2	MCS, MCD, C1, C3, C4	C2	C2
Lobular inflammation	0	27%	8%	0%	0%	19%	0%
	1	67%	4%	13%	13%	31%	31%
	2	2%	57%	64%	64%	50%	56%
	3	4%	31%	23%	23%	0%	13%
Significant		MCD, C2	MCS	N/S	MCS	N/S	N/S
Fibrosis	0	12.5%	0%	0%	0%	0%	0%
	1A	0%	18.8%	0%	0%	0%	0%
	1B	87.5%	62.5%	12.5%	14.3%	62.5%	37.5%
	1C	0%	0%	0%	0%	0%	0%
	2	0%	6.3%	62.5%	0%	37.5%	50%
	3	0%	12.5%	25%	85.7%	0%	12.5%
	4	0%	0%	0%	0%	0%	0%
Significant		MCD, C1, C2, C3, C4	MCS, C1, C2, C3, C4	MCS, MCD, C2, C3, C4	MCS, MCD, C1, C3, C4	MCS, MCD, C1, C2, C4	MCS, MCD, C1, C2, C3

Steatosis (0-3), portal inflammation (0-1), lobular inflammation (0-3) and fibrosis (0-4). Groups that are significantly different are listed below values, *p *< 0.05.

### Organ weight and body weight

Animals on the MCD and C1-C4 diet regimes had lower body weight compared to MCS animals (Table 5*p *< 0.001). Heart, kidney and pancreas weight were the same for all groups (data not shown). In contrast, liver weight represented a greater portion of body weight in the MCD and C1-C4 diet regimes compared to rats fed the MCS diet (Table 5*p *< 0.001). In addition, liver weight was significantly lower in the C2 diet regime (3.7 ± 0.1%) when compared to the MCD, C3 and C4 diet regimes, 4.4 ± 0.1%, 5.2 ± 0.2% and 4.1 ± 0.1%, respectively (Table 5*p *< 0.01). Average food intake over the duration of each dietary regime was in line with body weight; food intake did not differ between the cocoa regimes (Table 5).

**Table 5:** Biochemical parameters and measures of oxidative stress

	MCS	MCD	C1	C2	C3	C4
Food intake (g/pair/day)	24.4 ± 1.6	16.4 ± 0.5 MCS	13.4 ± 0.4 MCS	13.8 ± 0.6 MCS	12.4 ± 1.5 MCS	9.6 ± 0.5 MCS, MCD
Body weight (g)	283 ± 10	185 ± 4 MCS	192 ± 3 MCS	195 ± 7 MCS	188 ± 5 MCS	184 ± 5 MCS
Liver/body weight (%)	2.7 ± 0.1	4.4 ± 0.1 MCS	4.5 ± 0.3 MCS	3.7 ± 0.1 MCS, MCD	5.2 ± 0.2 MCS, C2	4.1 ± 0.1 MCS, C2
DHE (arbitrary units)	42.3 ± 2.1	71.6 ± 3.6 MCS	88.1 ± 1.0 MCS	87.9 ± 1.0 MCS	74.8 ± 3.7 MCS, C1, C2	88.8 ± 2.5 MCS, C3
Liver 8-OH-2dG (pg/ml)	192 ± 12	145 ± 5 MCS	265 ± 14 MCS, MCD	304 ± 12 MCS, MCD	205 ± 8 MCD, C1, C2	172 ± 7 C1, C2
Liver 8-isoprostane (pg/mg protein)	110 ± 12	155 ± 7 MCS	137 ± 9	163 ± 12 MCS	121 ± 5 MCD, C2	157 ± 7
Liver GSH (mg)	495 ± 64	1090 ± 156 MCS	120 ± 8 MCD	127 ± 9 MCD	106 ± 10 MCD	142 ± 6 MCD, C1, C3
RBC GSH (mg)	144 ± 8	177 ± 7 MCS	359 ± 26 MCS, MCD	432 ± 70 MCS, MCD	193 ± 15 MCS, C1, C2	120 ± 7 C1, C2
Glucose (mmol/L)	9.1 ± 0.4	6.8 ± 0.1 MCS	6.5 ± 0.2 MCS	6.0 ± 0.2 MCS	7.7 ± 0.1 MCS, C1, C2	6.6 ± 0.4 MCS
Triglycerides (mmol/L)	1.25 ± 0.05	0.99 ± 0.04 MCS	0.70 ± 0.02 MCD	0.66 ± 0.01 MCD, C1	0.71 ± 0.03 MCD	0.72 ± 0.01 MCD

Values are presented as mean ± SEM. Groups that are significantly different are listed below values, *p *< 0.05.

### Biochemical parameters

Circulating triglyceride levels were lower following consumption of the MCD diet when compared to the MCS diet (Table 5*p *< 0.001). This lower level was enhanced by the administration of cocoa supplement, resulting in a lower level of circulating triglycerides when compared to the MCD diet (Table 5*p *< 0.01). Duration of cocoa supplement enhanced this effect with C2 resulting in lower triglyceride levels when compared to C1 (Table 5*p *= 0.02).

Circulating glucose levels were also lower in animals on the MCD diet irrespective of cocoa supplementation when compared to MCS (Table 5*p *< 0.05). C1 and C2 resulted in significantly lower glucose when compared to C3 (Table 5*p *< 0.01).

### Measures of oxidative stress

Superoxide (DHE) levels were significantly higher in MCD fed animals compared to MCS fed animals (Table 5*p *< 0.001). Furthermore, superoxide levels were two fold higher in the C1, C2 and C4 groups when compared to animals fed the MCS diet (Table 5*p *< 0.001). C3 had the lowest superoxide levels when compared to the other cocoa groups (Table 5*p *< 0.01).

Liver GSH was twofold higher in MCD animals when compared to MCS diet fed animals (Table 5*p *< 0.01). Liver GSH was observed to be lower in all cocoa groups when compared to MCD (Table 5*p *< 0.001). In addition, C4 had significantly higher liver GSH when compared to the C1 and C3 diet regimes (Table 5*p *< 0.05).

Animals on the MCS diet had significantly lower RBC GSH when compared to those on the MCD and cocoa regimes (Table 5*p *< 0.01), with the exception of animals on the C4 diet regime. Animals on the C1 and C2 diet regimes had significantly higher RBC GSH levels, two fold and three fold respectively, when compared to MCS, MCD, C3 and C4 diet regimes (Table 5*p *< 0.01).

Liver 8-OH-2dG levels were significantly lower in MCD fed animals compared to MCS fed animals (Table 5*p *< 0.04). In contrast there was a significantly higher level of 8-OH-2dG in groups C1 and C2 compared to MCS and MCD fed animals (Table 5*p *< 0.001). Whereas, 8-OH-2dG levels in groups C3 and C4 were significantly lower than the levels observed in C1 and C2 (Table 5*p *< 0.001).

Liver 8-isoprostane levels were significantly higher in MCD fed animals and group C2 compared to MCS fed animals (Table 5*p *< 0.02). In contrast C3 has significantly lower levels of 8-isoprostane compared to MCD and C2 groups (Table 5*p *< 0.03).

### LFABP mRNA and Protein Expression

**Figure 2:**
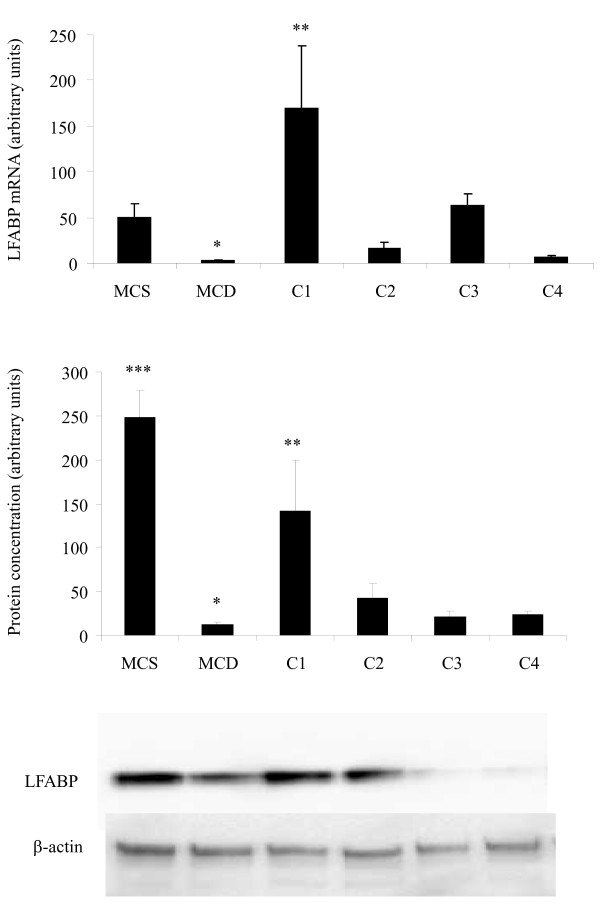
**Quantification of LFABP at the mRNA and protein levels**. (A) LFABP mRNA levels. (B) LFABP protein concentration. *Significant difference compared to MCS, *p *< 0.001. **Significant difference compared to MCD, *p *< 0.01. ***Significant difference compared to MCD, C2, C3 and C4, *p *< 0.001.

Lower levels of LFABP mRNA were observed following MCD diet consumption when compared to the MCS diet (Figure 2A, p < 0.001), but LFABP mRNA was 56 fold higher in animals fed the C1 diet when compared to the MCD diet (Figure 2A, p < 0.01). There was 20 fold lower LFABP protein levels in animals fed the MCD when compared to the MCS diet (Figure 2B, p < 0.001). The animals fed the MCS diet had higher levels of LFABP protein when compared to C2, C3 and C4 diet regimes (Figure 2B, p < 0.001). The C1 diet regime also showed higher levels of LFABP protein when compared to MCD (Figure 2B, p < 0.01).

### NOX mRNA and Protein Expression

**Figure 3:**
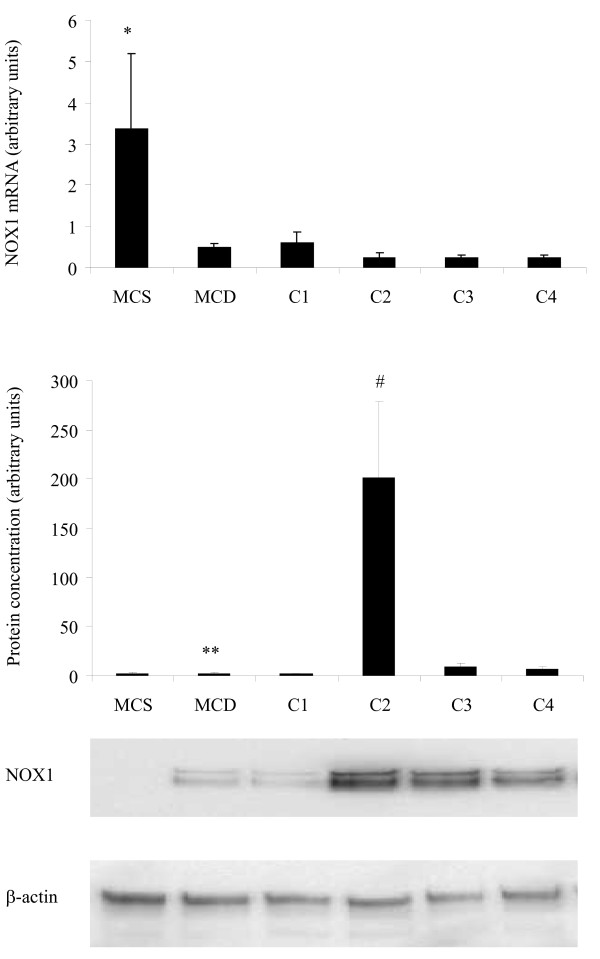
**Quantification of NOX1 at the mRNA and protein levels**. (A) NOX1 mRNA levels. (B) NOX1 protein concentration. *Significant difference compared to MCS, *p*≤0.05. **Significant difference compared to MCD, *p*≤0.03. #Significant difference compared to MCS, MCD, C1, C3 and C4, *p*≤0.01.

A fivefold depression of the NOX1 mRNA was observed following MCD diet consumption, with or without cocoa supplementation, when compared to the MCS diet (Figure 3A, p < 0.05). No difference was found in the mRNA levels of NOX2 and NOX4 between diet regimes. NOX1 protein levels were 20 fold higher in the C2 group when compared to MCS, MCD, C1, C3 and C4 diet regimes (Figure 3B, p < 0.01). Both C3 and C4 diet regimes had significantly higher NOX1 protein levels compared to the MCD diet (Figure 3B, p < 0.03).

## Discussion

The present study was carried out to determine if oxidative stress was associated with changes in the expression of LFABP and NOX in a rat model of non alcoholic steatohepatitis and whether cocoa supplementation attenuated those changes. The results indicate an association between the MCD diet and levels of LFABP in the development of NASH in a well established model of the disease. Levels of LFABP mRNA and protein were significantly lower in animals on the MCD diet in comparison to animals on the MCS diet. Suppression of LFABP may be another mechanism by which this diet causes an increased fat content in the liver in addition to impairing phosphatidylcholine synthesis [7]. Low levels of LFABP may lead to an inability of the hepatocyte to shuttle long chain fatty acids to different intracellular destinations for metabolism [22], resulting in higher levels of hepatic fat content in MCD animals as evident from the histological analysis (Figure 1; Table 4). Supplementation of MCD diet with cocoa in the C1 diet regime significantly increased levels of LFABP mRNA (Figure 2A), which we postulate leads to a restoration in trafficking of fatty acids within the hepatocyte; however this did not lead to a lower degree of observed steatosis (Table 4). Increased levels of LFABP may reduce oxidative damage by binding long chain fatty acids to its methionine residues [23]. Low levels of LFABP in MCD fed animals may therefore result in increased oxidative damage due to its ability to act as an endogenous antioxidant [9]. The increase in LFABP mRNA in the C1 diet regime (Figure 2A) showed a similar pattern at the protein level (Figure 2B). A decrease in LFABP may be linked to the liver's inability to cope with lipotoxicity, which is thought to contribute to NASH [24]. LFABP has been found to be upregulated in the presence of long chain fatty acids and has been directly implicated in hepatic regeneration [25]. This may be correlated to the effects of LFABP stimulation of PPAR-α to further increase LFABP mRNA. Findings in rat models indicate an increase in LFABP during hepatic regeneration, supporting the role of this protein in maintaining the integrity of the hepatocyte [25]. LFABP deregulation, as shown by an inverse relationship between the ratio of LFABP and fat content in the liver, has been correlated with obesity and type 2 diabetes in the Israeli sand rat [26]. This is further supported by a silencing of LFABP in patients with hepatocellular adenoma who had a mutation in the hepatocyte nuclear factor 1α, causing impaired trafficking of fatty acids, leading to steatosis [27]. Since LFABP is an abundant protein in hepatocytes, it may provide a major source of intracellular antioxidant activity. Purified LFABP has been tested for its antioxidant capacity [9] and is able to quench up to 66% of free radicals generated from superoxide. This is in agreement with our findings of lower LFABP being present at both the mRNA level (Figure 2A) and protein level (Figure 2B) in animals with MCD derived fatty liver disease in comparison to the animals fed the MCS diet. In addition, higher levels of superoxide fluorescence and 8-isoprostane were evident in the MCD fed animals as compared to the MCS fed animals (Table 3 and 5; Figure 1M and 1N), further supporting an inverse association between levels of LFABP and levels of oxidative stress. However, supplementation with cocoa in the C1 and C2 diet regimes resulted in higher superoxide and 8-OH-2dG levels when compared to MCS animals. This may be related to higher degree of observed steatosis in these groups (Table 4). Slightly lower superoxide and 8-OH-2dG levels were seen when animals were on the C3 diet regime. This C3 cocoa group had lower levels of steatosis when compared to MCD, C1 and C2 diet regimes. Further to this, lower levels of lobular inflammation and fibrosis were observed in these groups. It cannot be concluded that the higher levels of superoxide seen in the cocoa supplemented diets are as a result of the cocoa instead of the MCD, as the animals supplemented with cocoa were on the MCD diet longer than the MCD control group, dependent on the time of cocoa supplementation.

The quantification of mRNA detected differences in the levels of NOX1 mRNA expression, but no change observed in NOX2 and NOX4 mRNA expression between the different diet regimes. NOX1 mRNA expression levels were lower in all groups fed the MCD diet in comparison to those on the MCS diet (Figure 3A). The effect of the dietary regimes on NOX1 protein levels was different to that of mRNA expression levels (Figure 3B), indicating that NOX1 may be regulated at the protein level, rather than the gene level. Higher concentrations of NOX1 protein were observed in animals on the C2 diet regime. Gene knockout of gp91*^phox^*, a vital regulatory component of the assembly of NOX, showed no difference in the pathology of MCD induced NASH in mice compared to wildtype [11]. This would indicate that NOX generation of ROS is not a key factor in the development of MCD induced NASH, which is supportive of our findings in NOX mRNA expression. However, a link can be seen between NOX1 protein levels and presence of portal inflammation in animals on the C2 diet regime, with higher NOX1 levels measured and a greater proportion of portal inflammation observed in comparison to rats on the other diets. In alcoholic liver disease, mice fed ethanol via the Tsukamoto-French intragastric enteral method, NOX was found to increase ROS and activate NF-κB, which led to an increase in TNF-α in livers. This leads not only to an increase in oxidative damage but also an increase in synthesis of fatty acids causing hepatic damage [28].

Histological analysis of livers from rats fed the MCD diet showed greater steatosis in comparison to those on the MCS diet (Figure 1). Steatosis has been reported by others at week 2 of MCD feeding in rat livers [7]. The severity of steatosis was not observed to be less in any of the groups in which cocoa was added to the MCD diet, however there was a statistically significant lower degree of steatosis observed in livers of animals fed the C3 diet regime. It is extrapolated from this observation that the antioxidant properties of cocoa are more likely to affect levels of reactive oxidative species rather than hepatocyte fat content. This is supported by a lower level of ROS as determined by DHE staining and 8-OH-2dG in the C3 diet regime when compared to C1 and C2 diet regimes (Table 5). Antioxidants derived from cocoa may play a role in suppressing the activation of hepatic stellate cells to form fibrotic tissue, as fibrosis was not as severe in the animals on the C3 diet regime, a group which had lower scores for steatosis and lobular inflammation compared to other MCD and MCD/cocoa regimes (Table 4).

Circulating triglyceride levels were lower in the the MCD group compared to the control. However cocoa supplementation was associated with even lower circulating triglyceride levels (Table 5). Re-esterification of fatty acids into triglycerides has been described as a mechanism protecting the liver from lipotoxicity as inflammation, oxidative damage and fibrosis decrease [29]. Lower levels of circulating triglycerides (Table 5) found in our study are in line with increased severity of NAFLD as shown by increased steatosis scores in Table 4. The reduction in body weight on MCD possibly led to an increase in glucose being used as an energy source causing a reduction in the circulating levels of glucose (Table 5). The MCD diet has been previously reported to decrease glucose and improve insulin sensitivity whilst not having a dampening effect on the development of hepatic inflammation or fibrosis [29]. Although the MCD diet caused weight loss, liver weight increased as a result of higher fat content as seen in the histology of these samples (Figure 1; Table 4).

RBC GSH levels were significantly higher in the C1 and C2 groups (Table 5). This suggested that cocoa could be used to increase the availability of the reduced form of GSH to act as an antioxidant within RBC's and possibly the circulation. Liver GSH on the other hand was much lower in all cocoa supplemented animals when compared to those on the MCS and MCD diets. Low levels of this endogenous antioxidant in the cocoa supplemented animals may be due to the higher bioavailability of exogenous antioxidants derived from the cocoa. The accumulation of exogenous antioxidants from cocoa may therefore be beneficial in providing sufficient antioxidants to quench ROS in NASH. Our findings on hepatic GSH are not in agreement with most other studies which show a depletion of this endogenous antioxidant [7].

Despite the novel data presented from the current study there are limitations associated with the findings. Due to restrictions imposed by the institutional animal welfare committee it was not possible to include additional MCD fed rats for 80 and 108 days to match cocoa supplementation groups C1 - C4. Although pilot data indicated histologically the livers of rats fed the MCD diet are similar from 42 - 112 days, it cannot be excluded that the effects associated with cocoa supplementation in the liver are not to prolonged MCD feeding. It is possible, but unlikely, that the results observed following cocoa supplementation are not due to the antioxidants present in the cocoa, but rather the trace amounts of methionine and choline present in the cocoa. However if the trace amounts of methionine and choline present in the cocoa were responsible for the results observed it would be expected that data collected from the cocoa supplemented groups would more closely resemble the MCS group and not the MCD group. Finally although the MCD diet is a commonly used model of NASH there are a number of limitations associated with comparing the model to metabolic changes in human NAFLD/NASH [7]. These limitations include weight loss in rats fed the MCD diet, whereas NASH patients are typically overweight or obese [1,7]. The accumulation of fat within the liver of rats fed the MCD diet is due to a disruption of the export of hepatic lipids and subsequent lipotoxicity, unlike the human situation where the excessive hepatic fat import or storage is thought to occur [1,7].

## Conclusions

Our investigations indicate that the intracellular lipid transporter LFABP may play a key role in the establishment of MCD induced NAFLD and NASH not only by shuttling long chain fatty acids within the cell, but possibly by also acting as an antioxidant. Furthermore, the decreased levels of LFABP in the MCD model of NASH may suggest impairment in the functioning of LFABP in this disease. A cocoa rich diet is able to act as a rich source of exogenous antioxidants with no depletion of RBC GSH. However, this does not lead to lower hepatic superoxide and 8-OH-2dG levels. During the supplementation with the C1 diet regime, cocoa was associated with higher levels of LFABP compared to the MCD diet. There is depletion in the levels of NOX1 mRNA in animals on the MCD diet. NOX1 however is higher at the protein level in the animals on the C2 regime. The C2 regime simultaneously increased steatosis, portal inflammation and fibrosis, suggesting a possible role for NOX in inflammation and fibrosis in the rat NASH liver. As NASH develops in humans suffering from obesity and insulin resistance, further investigations into LFABP in the development of NASH in these patients is warranted. As fibrosis was less prominent in animals on the C3 diet regime, the role of antioxidants in influencing stellate cell activation and the development of fibrosis should be investigated.

## List of abbreviations

NAFLD: Non alcoholic fatty liver disease; NASH: Non alcoholic steatohepatitis; AST: Aspartate aminotransferase; ALT: Alanine aminotransferase; LFABP: Liver fatty acid binding protein; PPAR α: Peroxisome proliferator activated protein α; NOX: NADPH oxidase; NF-κβ: Nuclear factor κβ; TNF α: Tumor necrosis factor α; MCD: Methionine choline deficient; MCS: Methionine choline sufficient; H&E: Haematoxylin and Eosin; RBC: Red blood cell; GSH: Glutathione; DHE: Dihydroethidium; ROS: Reactive oxygen species; 8-OH-2dG: 8-hydroxy-2-deoxy Guanosine.

## Competing interests

The authors declare that they have no competing interests.

## Authors' contributions

MJ participated in the design of the study, carried out the analysis and interpretation of data and drafted the manuscript. KNA contributed to the interpretation of data and revised the manuscript. MJS-G carried out the analysis and interpretation of data and drafted the manuscript. MAM carried out the analysis and interpretation of data and drafted the manuscript. PAL participated in the design of the study, Carried of histological grading, contributed to the interpretation of data and revised the manuscript. All authors read and approved the final manuscript.
